# Long working hours and overweight and obesity in working adults

**DOI:** 10.1186/s40557-016-0110-7

**Published:** 2016-08-22

**Authors:** Byung-Mi Kim, Bo-Eun Lee, Hye-Sook Park, Young-Ju Kim, Young-Ju Suh, Jeong-youn Kim, Ji-Young Shin, Eun-Hee Ha

**Affiliations:** 1 National cancer control Institute, National Cancer Center, Goyang, South Korea; 2 Environmental Health Research Division, Environmental Health Research Department, National Institute of Environmental Research, Ministry of Environment, Incheon, South Korea; 3 Department of Preventive Medicine, School of Medicine, Ewha Womans University, Seoul, South Korea; 4 Department of Obstetrics and Gynecology, School of Medicine, Ewha Womans University, Seoul, South Korea; 5 Department of Biostatistics, Inha University Hospital and Center for Advanced Medical Education by BK21 project, College of Medicine, Inha University, Shinheung-dong 3ga, Chung-gu, Incheon, Korea; 6 Chronic Diseases Research Division, Korea Center for Disease Control and Prevention, Seoul, South Korea; 7 Worker Health Protection Division, Occupational safety and health Bureau, Ministry of labor Government Complex III, Seoul, South Korea

**Keywords:** Women, Long working hours, Age, Body mass index, Percentage body fat

## Abstract

**Background:**

Previous studies have identified a link between gender and the various risk factors associated with obesity. We examined obesity risk factors in working adults to identify the effects of differences in body mass index (BMI) and percentage body fat (PBF) between women and men.

**Methods:**

A total of 1,120 adults agreed to participate in the study. Data from 711 participants, including 411 women and 300 men, were analyzed. Multiple logistic regression analysis was used to estimate the effects of risk factors on obesity and being overweight. In addition, the least-squares (LS) means of both BMI and PBF were estimated by analysis of covariance (ANCOVA) in a generalized linear model.

**Results:**

Increases in BMI and PBF were significantly related to an age > 50 years and long working hours in women after compensating for confounding factors. Using the PBF criterion, the odds ratio (OR) of being overweight or obese in women > 50 years of age who worked for > 9 h a day was 3.9 (95% confidence interval [CI], 1.05–11.00). For BMI, women who were > 50 years of age and worked for > 9 h a day were 3.82 times (95% CI, 1.31–11.14) more likely to be overweight or obese than those who were < 50 years of age and worked for < 9 h a day.

**Conclusion:**

Obesity in working adults was associated with > 50 years of age and long working hours in women. Further studies are needed to investigate the underlying mechanisms of this relationship and its potential implications for the prevention and management of excess weight and obesity.

## Background

Obesity, the accumulation of excess body fat, is recognized as an independent risk factor for a variety of chronic diseases, such as hypertension, ischemic heart disease, fatty liver, cholelithiasis, hyperlipidemia, diabetes, and osteoarthritis. Obesity also affects the development of some types of cancer in men (prostate and colorectal cancers), as well as breast and ovarian cancers in women. Thus, obesity is an important issue in public health [1].

The prevalence of obesity has been increasing in several countries [2–5]. The Korea National Health and Nutrition Examination Survey has recently reported that the rate of obesity in adults has been steadily increasing each year. In men, there was a significant increase over time in prevalence of obesity (26.8 % for KNHANES I (1998), 37.6 % for KNHANES III (2005), and 38.1 % for KNHANES V (2010–2012); P for trend < 0.0001), and these increases were consistent across all age groups. However, the prevalence of obesity among men has remained stable since KNHANES III. In contrast, the prevalence of obesity in women decreased over time, but the decrease was not significant (P for trend = 0.14) [6]. When comparing the incidence of obesity by gender and age group, men had the highest incidence of obesity in their 40s–50s, while women had the highest incidence in their 50s–60s [6]. 

Previous studies have reported that obesity is more prevalent in women than men mainly because the distribution of a woman’s body fat changes depending on the hormone cycle [7]. The literature suggests that the accelerated increase in total body fat experienced by many middle-aged women is not due to the general aging process, but is a phenomenon triggered by menopause and the withdrawal of estrogens [8].

The determinants of obesity have been shown to be multifactorial and gender-specific [9]. In particular, long working hours have been found to interrupt the recovery of workers from overwork, inhibit performance of regular exercise, and decrease sleep duration [10]. According to the Organization for Economic Cooperation and Development (OECD), the Republic of Korea had the longest working hours until 2007, and the second longest working hours from 2008 to 2011 [11]. An association between long working hours and obesity has been reported in several countries, including Hong Kong, Australia and Finland [12–14].

Although studies showed a significant effects of long working hours on male obesity, the association between number of working hours and obesity in the female population was inconclusive. 14.8% more female than male workers are employed in the informal sector [15]. With increasing participation in employment and exposure to poorer labor environments among female compared to male workers, it is becoming more important to study the risk factors in the labor environment on obesity in female workers. However, to the best of our knowledge, few studies have investigated this topic. The main purpose of this study was to explore the relationships between number of working hours and excess weight and obesity among working adults in Seoul, Korea.

## Methods

### Study design and subjects

All respondents were > 40 years of age (range 40 to 78) and had a medical examination at Ewha Womans University Hospital (one of the participants in the Korea Health Examinees Cohort study from June 2005 to March 2006). All participants were given a full written and verbal explanation of the project and each subject provided written consent before enrollment. The participants completed a questionnaire, which included questions about socioeconomic and lifestyle factors. One thousand one hundred twenty subjects responded that they currently had a job. Four hundred nine participants whose data showed missing values for major variables, such as the body mass index (BMI) and percent body fat (PBF), were excluded so that a total of 711 participants (411 women and 300 men) were included in the final analysis, comprising 63.5% of the original group of study subjects (Table 1). The study was approved by the Institutional Review Board (IRB) of Ewha Womans University Hospital in Seoul, South Korea.

**Table 1:** Characteristics of study subjects by gender^a^

Variable	N (%), Mean ± SD	
	Women(*N* = 411)	Men(*N* = 300)
General characteristics		
Age group		
40–49	220(53.5)	141(47)
50–59	123(37.5)	111(37)
≥ 60	57(17.4)	48(16)
Marital status		
Unmarried	10(2.5)	8(2.7)
Married	367(91.5)	277(93.9)
Divorced/Widow/Widower	24(6.0)	10(3.4)
Educational level		
Low	200(48.7)	129(43)
Middle	191(46.5)	136(45.3)
High	20(4.9)	35(11.7)
Household income (US$/Month)		
< 2,000	21(5.8)	18(6.3)
2,000–3,000	18(5)	17(6.0)
≥ 3,000	321(89.2)	249(87.7)
Smoking		
Non-smoker	396(96.3)	86(28.7)
Smoker	15(3.7)	214(71.3)
Alcohol consumption		
Non-drinking	284(69.1)	54(18)
Drinking	127(30.9)	246(82)
Anthropometric characteristics		
BMI(kg/m^2^)^*^	23.2 ± 2.9	24.5 ± 2.7
Prevalence of overweight(%)^+^	102(24.8 %)	85(28.3 %)
Prevalence of obesity(%)^+^	97 (23.6 %)	134(44.7 %)
PBF(%)^*^	27.3 ± 5.3	23.9 ± 4.5
Prevalence of overweight(%)^+^	156 (38.0 %)	115 (38.3 %)
Prevalence of obesity(%)^+^	48 (11.7 %)	136(45.3 %)
Lean body mass(kg)^*^	36.9 ± 3.5	48.9 ± 4.5

^*^Values are Mean ± SD

^+^Abbreviations. BMI: Body mass index (Cut-off limits of overweight: 23 ≤ BMI (kg/m^2^) < 25, Cut-off limits of obesity: BMI (kg/m^2^) ≥25), PBF: Percent body fat (Cut-off limits of overweight: 20 = <PBF (%) < 25 in men and 30 ≤ PBF (%) < 35 in women, Cut-off limits of obesity: men’s PBF (%) ≥ 25; women’s PBF (%) ≥ 35)

^a^Differences between sexes: *P < 0.05 obtained by students *t*-test, **P < 0.05 obtained by *χ* ^2^-test

### Anthropometric measurements

Anthropometric and body composition measurements were performed by two experienced nurses. Height was measured Using an ultrasonic height meter (FA-92H; Panics, Korea) and weight was measured without shoes and heavy clothing using a body composition analyzer. The height and weight measurements were recorded to the nearest 0.1 cm and 0.1 kg, respectively.

For the body composition analysis, the PBF and muscle mass were measured using a body composition analyzer (Zeus 9.9 Jawon medical Co. Ltd., Seoul, Korea, 2013) utilizing a bioelectrical impedance analysis (BIA), which is widely used in obesity clinics for the assessment of body composition and does not require any exposure to radiation.

### Case definition

Categories were selected according to the standards of the World Health Organization (WHO) Western Pacific Regional Office proposal [9], which is widely used in Korea to define individuals that are of healthy weight (BMI 20.0–23.0 kg/m2), overweight (23–24.9 kg/m2), and obese (BMI ≥ 25 kg/m2). Men with PBFs < 20 %, 20–24 %, and ≥ 25 % were classified as healthy weight, overweight, or obese, respectively, while women were classified as healthy weight, overweight, or obese with PBFs of <30 %, 30–34 %, and ≥35 %, respectively [16].

### Socioeconomic and lifestyle factors

We included socioeconomic factors, including age, marital status (unmarried, married, and divorced/widow/widower), educational level (<12, 12–16, and ≥16 years) and monthly household income (<200, 200–300, and ≥300 × 104 won) in the questionnaire. For lifestyle factors, we investigated smoking (non-smoker or smoker), alcohol consumption (drinker or non-drinker), regular exercise (yes or no), average number of working hours per day, average number of sleeping hours per day, and average occupational sitting time. Occupational sitting times were dichotomized at the median value of 4 h/day and categorized as: <4 h/day and ≥4 h/day.

### Statistical analysis

Descriptive statistics for general characteristics and anthropometric measures were calculated separately for each gender. To analyze factors related to obesity and being overweight according to gender, we performed a χ2 test or an analysis of variance (ANOVA). We used multiple logistic regression to determine the association between obesity-related risk factors and BMI and PBF adjusted for confounding factors except for the targeted variable itself. The least squares means (LS means) of BMI and PBF according to the risk factors were estimated by analysis of covariance (ANCOVA) in the generalized linear model. All reported *p* values were calculated by two-sided tests at a significance level of 5 %. All analyses were performed using SAS (version 9.3; SAS Institute, Cary, NC, USA).

## Results

Table 1 lists the characteristics of the study participants by gender. Approximately one-half of the study subjects were in their 40s and were college graduates. The percentages of women who smoked cigarettes and drank alcohol were 3.7 % and 30.9 %, respectively. Men had a higher proportion of being obesity (44.7 %) than women (23.6 %, p < 0.05). The proportion of obesity based on the PBF was higher in men (45.3 %) than in women (11.7 %).

Table 2 shows the proportion of each gender that was overweight or obese individuals according to socioeconomic and lifestyle factors. In women, age and a low level of education were significantly related with being overweight, as based on BMI and PBF. In addition, the proportion of overweight individuals was significantly higher in women who worked > 9 h a day. In contrast, in men none of the socioeconomic and lifestyle factors were associated with being overweight.

**Table 2:** Prevalence of socioeconomic and lifestyle characteristics for being overweight or obese based on BMI and PBF by gender

	BMI (kg/m^2^)				PBF (%)			
	Women		Men		Women		Men	
Variable	Controls^*^	Cases^+^	Controls^*^	Cases^+^	Controls^*^	Cases^+^	Controls^*^	Cases^+^
	(*N* = 207)	(*N* = 204)	(*N* = 81)	(*N* = 219)	(*N* = 207)	(*N* = 204)	(*N* = 49)	(*N* = 251)
Age group								
40–49	126 (59.4)	94 (47.2)	34 (42)	107 (42)	132 (63.8)	88 (43.1)	25 (51)	116 (46.2)
50–59	69 (32.6)	79 (39.7)	31 (38.3)	80 (38.3)	67 (32.4)	81 (39.7)	17 (34.7)	94 (37.5)
≥ 60	17 (8)	26 (13.1)	16 (19.8)	32 (19.8)	8 (3.9)	35 (17.2)	7 (14.3)	41 (16.3)
	*p* = 0.01		*p* = 0.21		*p* < 0.0001		*p* = 0.55	
Marital status								
Unmarried	5 (2.4)	5 (2.6)	3 (3.8)	5 (2.3)	5 (2.5)	5 (2.5)	1 (2)	7 (2.9)
Married	191 (92.3)	176 (90.7)	74 (92.5)	203 (94.4)	187 (92.6)	180 (90.5)	45 (91.8)	232 (94.3)
Divorced/Widow/Widower	11 (5.3)	13 (6.7)	3 (3.8)	7 (3.3)	10 (5)	14 (7)	3 (6.1)	7 (2.9)
	*p* = 0.67		*p* = 0.79		*p* = 0.48		*p* = 0.29	
Educational level								
Low	89 (42)	111 (55.8)	36 (44.4)	93 (42.5)	89 (43)	111 (54.4)	23 (46.9)	106 (42.2)
Middle	110 (51.9)	81 (40.7)	40 (49.4)	96 (43.8)	104 (50.2)	87 (42.7)	22 (44.9)	114 (45.4)
High	13 (6.1)	7 (3.5)	5 (6.2)	30 (13.7)	14 (6.8)	6 (2.9)	4 (8.2)	31 (12.4)
	*p* = 0.005		*p* = 0.28		*p* = 0.01		*p* = 0.40	
Income (US$/Month)								
< 2,000	12 (6.6)	9 (5.1)	4 (5)	14 (6.9)	11 (6.1)	10 (5.6)	2 (4.1)	16 (6.8)
2,000-3,000	8 (4.4)	10 (5.6)	5 (6.3)	12 (5.9)	7 (3.9)	11 (6.2)	2 (4.1)	15 (6.4)
≥ 3,000	162 (89)	159 (89.3)	71 (88.8)	178 (87.3)	163 (90.1)	158 (88.3)	45 (91.8)	204 (86.8)
	*p* = 0.73		*p* = 0.63		*p* = 0.81		*p* = 0.35	
Smoking								
Non-smoker	207 (97.6)	189 (95)	25 (30.9)	61 (27.9)	202 (97.6)	194 (95.1)	15 (30.6)	71 (28.3)
Smoker	5 (2.4)	10 (5)	56 (69.1)	158 (72.2)	5 (2.4)	10 (4.9)	34 (69.4)	180 (71.7)
	*p* = 0.24		*p* = 0.71		*p* = 0.28		*p* = 0.88	
Alcohol consumption								
Non-drinking	151 (71.2)	133 (66.8)	14 (17.3)	40 (18.3)	146 (70.5)	138 (67.7)	10 (20.4)	44 (17.5)
Drinking	61 (28.8)	66 (33.2)	67 (82.7)	179 (81.7)	61 (29.5)	66 (32.4)	39 (79.6)	207 (82.5)
	*p* = 0.39		*p* = 0.98		*p* = 0.60		*p* = 0.78	
Working hours								
< 9 h/day	193 (93.7)	169 (86.7)	47 (58.8)	146 (67.6)	189 (93.6)	173 (86.9)	29 (60.4)	164 (66.1)
≥ 9 h/day	13 (6.3)	26 (13.3)	33 (41.3)	70 (32.4)	13 (6.4)	26 (13.1)	19 (39.6)	84 (33.9)
	*p* = 0.03		*p* = 0.20		*p* = 0.04		*p* = 0.55	
Occupational sitting time								
< 4 h/day	152 (71.7)	132 (66.3)	28 (34.6)	74 (33.8)	149 (72)	135 (66.2)	14 (28.6)	88 (35.1)
≥ 4 h/day	60 (28.3)	67 (33.7)	53 (65.4)	145 (66.2)	58 (28)	69 (33.8)	35 (71.4)	163 (64.9)
	*p* = 0.28		*p* = 1.00		*p* = 0.24		*p* = 0.48	
Regular exercise								
No	89 (42.4)	73 (36.9)	34 (42.5)	84 (38.7)	79 (38.5)	83 (40.9)	19 (38.8)	99 (39.9)
Yes	121 (57.6)	125 (63.1)	46 (57.5)	133 (61.3)	126 (61.5)	120 (59.1)	30 (61.2)	149 (60.1)
	*p* = 0.30		*p* = 0.65		*p* = 0.70		*p* = 1.00	
Sleeping hours								
> 9 h/day	135 (65.5)	146 (75.7)	59 (72.8)	157 (72)	132 (65.4)	149 (75.6)	35 (71.4)	181 (72.4)
**≤** 9 h/day	71 (34.5)	47 (24.4)	22 (27.2)	61 (28)	70 (34.7)	48 (24.4)	14 (28.6)	69 (27.6)
	*p* = 0.04		*p* = 1.00		*p* = 0.03		*p* = 1.00	

^*^Controls. BMI: Body mass index (Cut-off limits of overweight: BMI (kg/m^2^) <25, PBF: Percent body fat (Cut-off limits of overweight: men’s BF (%) < 20; women’s BF (%) < 30)

^+^Cases. BMI: Body mass index (Cut-off limits of overweight: BMI (kg/m^2^) ≥25, PBF: Percent body fat (Cut-off limits of overweight: men’s BF (%) ≥ 20; women’s BF (%) ≥ 3

Working hours, occupational sitting time and Sleeping time were dichotomized at the median

The results obtained from the logistic regression analyses for BMI and PBF are presented in Table 3. The ORs of being overweight or obese in women for both BMI and PBF were significantly higher in older women. Women with a lower educational level were more likely to be overweight or obese. Among women who worked >9 h a day, the risk for becoming overweight or obese increased according to both the BMI and PBF criteria after adjusting for confounding factors such as age, educational level, smoking, alcohol consumption, number of hours worked, sitting time at work, and number of sleep hours. Based on the BMI and PBF, the ORs for obesity attributable to long working hours were 2.42 (95 % CI, 1.05–5.57) and 2.50 (95 % CI, 1.07–5.79), respectively.

**Table 3:** Odds ratio and 95 % Confidence intervals of overweight or obese based on BMI and PBF by socioeconomic and lifestyle characteristics for women and men

	BMI (kg/m^2^)				PBF (%)			
	Women		Men		Women		Men	
Variable	Crude OR	Adjusted OR^*^	Crude OR	Adjusted OR^*^	Crude OR	Adjusted OR^*^	Crude OR	Adjusted OR^*^
	(95 % CI)	(95 % CI)	(95 % CI)	(95 % CI)	(95 % CI)	(95 % CI)	(95 % CI)	(95 % CI)
Socio-demographic factors								
Age group								
40–49	1	1	1	1	1	1	1	1
50–59	1.54	1.47	0.82	0.66	1.81	1.96	1.19	1.41
	(1.01–2.33)	(0.89–2.42)	(0.47–1.45)	(0.34–1.28)	(1.19–2.76)	(1.18–3.24)	(0.61–2.34)	(0.66–3.02)
60+	2.05	2.49	0.64	0.48	6.56	7.37	1.26	1.65
	(1.05–4.00)	(1.16–5.33)	(0.31–1.30)	(0.21–1.11)	(2.91–14.81)	(3.06–17.76)	(0.51–3.14)	(0.57–4.76)
Marital status								
Unmarried	1	1	1	1	1	1	1	1
Married	0.92	0.74	1.65	1.65	0.96	1.41	0.74	0.94
	(0.26–3.24)	(0.17–3.18)	(0.38–7.06)	(0.35–7.71)	(0.27–3.38)	(0.30–6.65)	(0.09–6.13)	(0.11–8.35)
Divorced, Widow/Widower	1.18	0.51	1.4	0.83	1.4	0.75	0.33	0.14
	(0.27–5.18)	(0.09–2.89)	(0.20–10.03)	(0.09–7.49)	(0.32–6.16)	(0.12–4.7)	(0.03–4.04)	(0.01–2.09)
Educational level								
Low	1	1	1	1	1	1	1	1
Middle	0.59	0.72	0.93	0.87	0.67	0.87	1.12	1.29
	(0.40–0.88)	(0.45–1.16)	(0.55–1.58)	(0.48–1.59)	(0.45–1.00)	(0.54–1.41)	(0.59–2.14)	(0.64–2.61)
High	0.43	0.20	2.32	2.60	0.34	0.21	1.68	1.80
	(0.17–1.13)	(0.05–0.78)	(0.84–6.45)	(0.81–8.36)	(0.13–0.93)	(0.05–0.85)	(0.54–5.23)	(0.54–6.02)
Income (US$/Month)								
< 2,000	1	1	1	1	1	1	1	1
2,000–3,000	1.67	1.72	0.69	0.42	1.73	1.63	0.94	0.99
	(0.47–5.93)	(0.41–7.31)	(0.15–3.15)	(0.08–2.33)	(0.48–6.2)	(0.37–7.20)	(0.12–7.52)	(0.11–9.07)
≥ 3,000	1.31	1.29	0.72	0.40	1.07	0.82	0.57	0.41
	(0.54–3.19)	(0.48–3.48)	(0.23–2.25)	(0.10–1.57)	(0.44–2.58)	(0.30–2.24)	(0.13–2.55)	(0.08–2.03)
Lifestyle factors								
Smoking								
Non-smoker	1	1	1	1	1	1	1	1
Smoker	2.19	1.87	1.16	1.47	2.08	1.86	1.12	1.25
	(0.74–6.52)	(0.55–6.39)	(0.66–2.02)	(0.77–2.81)	(0.70–6.20)	(0.54–6.43)	(0.57–2.18)	(0.58–2.67)
Alcohol consumption								
Non-drinking	1	1	1	1	1	1	1	1
Drinking	1.23	1.39	0.94	0.91	1.15	1.44	1.21	1.70
	(0.81–1.87)	(0.85–2.25)	(0.48–1.83)	(0.43–1.96)	(0.75–1.74)	(0.88–2.37)	(0.56–2.60)	(0.73–3.93)
Working hours								
< 9 h/day	1	1	1	1	1	1	1	1
≥ 9 h/day	2.28	2.42	0.68	0.46	2.19	2.50	0.78	0.63
	(1.14–4.59)	(1.05–5.57)	(0.40–1.16)	(0.24–0.87)	(1.09–4.39)	(1.07–5.79)	(0.41–1.48)	(0.30–1.32)
Occupational sitting time								
< 4 h/day	1	1	1	1	1	1	1	1
≥ 4 h/day	1.29	0.96	1.04	1.16	1.26	0.98	0.85	0.91
	(0.85–1.96)	(0.53–1.76)	(0.61–1.77)	(0.63–2.12)	(0.78–2.06)	(0.53–1.83)	(0.46–1.57)	(0.45–1.85)
Regular exercise								
No	1	1	1	1	1	1	1	1
Yes	1.26	1.04	1.17	1.31	0.91	0.81	0.95	0.89
	(0.85–1.88)	(0.65–1.66)	(0.70–1.97)	(0.74–2.33)	(0.61–1.35)	(0.5–1.32)	(0.51–1.79)	(0.46–1.74)
Sleeping hours								
> 9 h/day	1	1	1	1	1	1	1	1
**≤** 9 h/day	0.61	0.66	1.04	1.05	0.61	0.65	0.95	0.79
	(0.40–0.95)	(0.40–1.09)	(0.59–1.85)	(0.56–1.99)	(0.39–0.94)	(0.39–1.10)	(0.48–1.88)	(0.39–1.63)

^*^Adjusted for confounding factors except for the targeted variable itself. The confounding factors involve age, educational level, smoking, alcohol consumption, working hours, daily occupational sitting time, and sleeping hours (h/day)

**Fig. 1:**
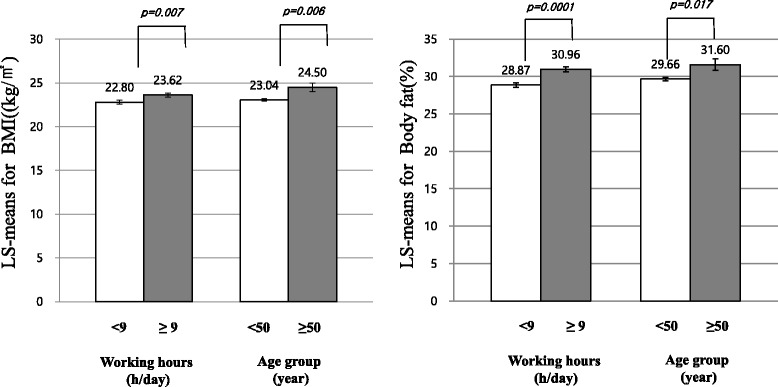
Levels of BMI and PBF by gender according to daily working hours and age groups. Levels were adjusted for educational level, smoking, alcohol consumption, working hours, and sleeping time (hours/day). Working hours and age group were dichotomized at the median. LS-means: least squares means

 Figure 1 compares the estimated LS means of BMI and PBF by gender according to age and number of working hours. BMI and PBF significantly increased with age and number of working hours in women after adjusting for education, smoking, alcohol consumption, and the average number of sleep hours a day.

The ORs of being overweight or obese for the combined effects of age and long working hours at work are given in Table 4. Using the PBF criterion, the OR of being overweight or obese in women > 50 years of age who worked > 9 h a day was 3.9 (95 % CI, 1.05–11.00). For BMI, women > 50 years of age who worked > 9 h a day were 3.56 times (95 % CI, 1.03–12.37) more likely to be overweight or obese than those who were <50 years of age and worked < 9 h a day.

**Table 4:** Combined effects of age and daily working hours for overweight or obesity based on BMI and PBF criterion in women

Category	N (%)	Odds ratios (95 % Confidence Intervals)			
		BMI		PBF	
		Crude	Adjusted^*^	Crude	Adjusted^*^
Group1 (Age < 50 and working hours < 9)	263 (37.73)	1	1	1	1
Group2 (Age ≥ 50 and working hours < 9)	292 (41.89)	1.69(1.11–2.56)	1.68(1.04–2.73)	2.66(1.67–3.91)	2.87(1.75–4.69)
Group3 (Age < 50 and working hours ≥ 9)	87 (12.48)	2.39(0.94–6.03)	2.34(0.77–7.11)	2.60(1.32–8.90)	2.73(1.19–12.06)
Group4 (Age ≥ 50 and working hours ≥ 9)	55 (7.89)	3.82(1.31–11.14)	3.56(1.03–12.37)	3.43(1.23–9.54)	3.90(1.05–11.00)

^*^Adjusted for confounding factors except for the targeted variable itself. The confounding factors involve age, educational level, smoking, alcohol consumption, working hours, daily occupational sitting time, and sleeping hours (h/day). Working hours and age group were dichotomized at the median

## Discussion

We found associations among obesity (BMI and PBF), age > 50 years, and long work hours in female workers. Women > 50 years of age had a higher incidence of obesity and being overweight than other age groups. Data from large population studies have shown that mean body weight and BMI gradually increase during most of the adult life and reach peak values at 50–59 years of age in women [17–20]. This may be explained by women’s weight rapidly increasing as their physical activity and energy consumption decrease following menopause [21]. Obesity may also exaggerate unfavorable lipid profiles in aging and menopausal women. Some studies have reported a more frequent age-related increase in the prevalence of being overweight or obese [7, 22] in females than in males [23] In addition, studies have reported a greater increase in fat mass and waist circumference or abdominal skinfold thickness in postmenopausal compared to premenopausal women [23–25] An increase in abdominal lipoprotein lipase activity is observed after the withdrawal of estrogens, which leads to the local elevation of free fatty acids and the accumulation of abdominal fat [26, 27].

Our data showed that higher mean number of daily working hours was associated with higher BMI and PBF. A significant association was identified between obesity and working >9 h a day among females. A previous study in Korea also reported a relationship between long working hours and obesity, but the relationship was not statistically significant [28].

The pathology of obesity as an imbalance between energy intake, multifactor, and energy expenditure is the most important mechanism inducing obesity. Inadequate working conditions linked to the induction of the stress response, and poor lifestyle factors such as long working hours, may increase the risk for obesity [29], When stressful events affect the hypothalamic pituitary adrenal (HPA) axis glucocorticoids are released as end hormones. Glucocorticoids inhibit the positive effects of the growth and thyroid hormones on lipolysis and muscle anabolism [30]. Poor lifestyle factors have also been linked to hormonal factors such as reduced levels of leptin, [31] an adipokine strongly linked to appetite and fat storage that increases the risk for obesity [32]. Hence, the association between long working hours and obesity identified in the current study might be caused by the induction of a stress response as a reaction to working long hours and its associated metabolic effects [33].

Asian women generally assume much of the responsibility for housework. This cultural norm increases stress among female workers, who have less time to complete household duties after long hours spent at the workplace. A longitudinal study reported that women are more vulnerable to anxiety due to overwork than men [34]. The results of the current study suggest that working long hours induces chronic stress, which can increase the risk for obesity in Asian women. Therefore, attention should be paid to the working hours of women to reduce their risk for obesity. Several researchers have suggested that long working hours can lead to poor sleeping habits and less time for exercise and maintaining a balanced diet [35]. which are strongly linked to obesity and increased risk for cardiovascular diseases.

Our study had various strengths as well as limitations. The cross-sectional analyses did not allow us to determine the temporality of the associations, and the data were old. In addition, physical activity and diet may be important confounding variables in the association between obesity and number of working hours, which were not considered in this study due to a lack of data. In other words, obesity and its related health conditions can decrease work performance, and an obese worker might need more time to complete the job demand compared to a healthy worker [13, 36, 37]. This could have biased our results. Hence, careful consideration is needed to interpret our results accurately, and an appropriate prospective cohort study is needed to elucidate this causal relationship. However, we used PBF as an indicator of obesity and employed the BIA method to precisely diagnose obesity. BIA is a widely used method for estimating body composition and has increasingly been used in Korea [38]. The technology is relatively simple, quick, and noninvasive. It is currently used in such diverse settings as the offices of private clinicians, health clubs, hospitals, and across a spectrum of ages, body weights, and disease states. Despite a general perception among the public that BIA measures body fat, the technology actually determines the electrical impedance of body tissues, which provides an estimate of total body water.

## Conclusions

In conclusion, obesity was associated with aging and long working hours in female workers in Korea. Further studies are needed to investigate the underlying mechanisms of this relationship and its potential implications for the prevention and management of obesity.

## Abbreviations

ANCOVA: Analysis of covariance
BIA: Bioelectrical Impedance Analysis
BMI: Body Mass Index
IRB: Institutional Review Board
LS means: Least Squares means
MONICA: Monitoring Trends and Determinants in Cardiovascular Disease
PBF: Percentage body fat
WHO: World Health Organizations
